# Comparison of therapy with β-lactam/β-lactamase inhibitor combinations or carbapenems for bacteraemia of nonurinary source caused by ESBL-producing* Escherichia coli* or *Klebsiella pneumoniae*

**DOI:** 10.1186/s12941-021-00471-6

**Published:** 2021-09-06

**Authors:** Hong Luo, Yanping Xiao, Yaping Hang, Yanhui Chen, Hongying Zhu, Xueyao Fang, Xingwei Cao, Shan Zou, Xiaoyan Hu, Jianqiu Xiong, Qiaoshi Zhong, Longhua Hu

**Affiliations:** 1 Department of Jiangxi Provincial Key Laboratory of Medicine, Clinical Laboratory of the Second Affiliated Hospital of Nanchang University, Mingde Road No. 1, Nanchang, 330006 Jiangxi People’s Republic of China; 2 Department of Nursing, The Second Affiliated Hospital of Nanchang University, Mingde Road No. 1, Nanchang, 330006 Jiangxi People’s Republic of China

**Keywords:** ESBL, *Escherichia coli*, *Klebsiella pneumoniae*, BSI, Nonurinary source, BLICs, Carbapenems

## Abstract

**Background:**

Extended-spectrum β-lactamase (ESBL)-producing Enterobacteriaceae has become a public health concern. This study aimed to compare the clinical outcomes of patients with nonurinary source bacteraemia caused by ESBL-producing *Escherichia coli* (*E. coli*) or *Klebsiella pneumoniae* (ESBL-producing EK) receiving *β*-lactam/*β*-lactamase inhibitor combinations (BLICs) versus carbapenem treatment and assess the risk factors of mortality with these two drugs.

**Methods:**

We conducted a retrospective single-centre study of adult hospitalised patients with ESBL-producing EK bloodstream infection (BSI) from nonurinary source at our centre over a 4-year period. One hundred and eighty patients who received BLICs or carbapenems were included in the analysis. The outcome variables were 14-day treatment failure and 30-day mortality. For more reliable results, propensity score analysis was performed to compare the efficacy of the two drugs and analyse their risk factors for 30-day mortality.

**Results:**

Out of 180 patients, 114 received BLICs, and 66 received carbapenem therapy. Compared to carbapenem-treated patients, those treated with BLICs were older and had higher age-adjusted Charlson comorbidity index, but they had shorter stay in the hospital. Additionally, their Pitt bacteraemia score, SOFA score, rate of leukaemia, and immune compromise were lower. After propensity score matching (PSM), the baseline characteristics of patients in the two treatment groups were balanced. BLICs were associated with a higher 14-day treatment failure rate (20.6%, 13/63) than carbapenems (16.3%, 7/43), although the difference was not significant in either univariate analysis (P = 0.429) or multivariate analysis (P = 0.122). And the 30-day mortality rate in BTG (11.1%, 7/63) and CTG (11.6%, 5/43) did not significantly differ (univariate analysis, P = 0.926; multivariate analysis, P = 0.420). In the multivariate analysis, after PSM, leukaemia was the only independent predictor of mortality in both BTG and CTG.

**Conclusions:**

Our study showed that BLICs had higher 14-day treatment failure rate compared with carbapenems, although there were no statistically significant differences because of the small number of patients, therefore, further evaluation of the efficacy of BLICs is needed.

## Background

Despite the enormous strides in medical science over the past few centuries, bloodstream infection remains a growing public health threat worldwide [1]. Timely and appropriate antimicrobial therapy is crucial for the treatment of patients with bacteraemia [2, 3]. In patients with BSI caused by ESBL-producing bacteria, the choice of antibiotics is limited. Carbapenems owing to their exceptionally broad spectrum of activity, structural stability to almost all β-lactamases, and proven clinical efficacy, have been regarded as the gold standard for the treatment of ESBL producers, even when active in vitro to other antibiotics has been proven [4, 5]. However, increased consumption of carbapenems creates a selection pressure, leading to the emergence of carbapenem-resistant bacteria, which are associated with alarming mortality rates [6, 7]. Utilising carbapenem-sparing agents for the therapy of ESBL-producing Enterobacteriaceae is an effective strategy to reduce the utilisation of carbapenems and the associated downstream effects of carbapenem overutilization [8]. Therefore, seeking and re-evaluating alternative regimens for carbapenems is a matter of great urgency.

BLICs are one of the most common agents used in patients with Enterobacteriaceae bloodstream infections in clinical practice [9]. Several published studies have compared the efficacy of BLICs and carbapenems for treating bloodstream infections (BSI) caused by ESBL-producing Enterobacteriaceae, with variable results [5, 10–13].

It has been suggested that the inoculum and the source of infection has notable impact on the efficacy of a specific antibiotic [14, 15], which may explain some conflicting results in different studies. In fact, compared with bacteraemia from urinary source, those nonurinary source bacteraemia are higher inoculum infection and usually more severe [16]. However, the majority of existing studies do not distinguish the source BSI [12, 17–19], and bacteraemia in some participants were urinary source, which may lead to the overestimation of the efficacy of BLICs, because, according to current data, there is support for the use of BLICs for patients with ESBL-producing Enterobacteriaceae bacteraemia from a urinary source [14–16, 20, 21]. For BSI derived only from nonurinary source, a relatively high-inoculum infection, there are limited investigations.

Under these premises, the objective of this study was to investigate whether BLICs were as effective as carbapenems in hospitalised patients with nonurinary source bacteraemia caused by ESBL-producing EK, and to assess the risk factors of mortality with these two drugs.

## Methods

### Study design and patients

A single-centre, retrospective cohort study was performed from January 1, 2016 to December 31, 2019 at the Second Affiliated Hospital of Nanchang University, a 2475-bed tertiary hospital located in Jiangxi Province, China. The clinical information of patients was collected through the database of the Laboratory Information System (LIS) and Hospital Information System (HIS) in our hospital.

For this analysis, adult (age ≥ 18) patients were included if they diagnosed with monomicrobial ESBL-producing EK bacteraemia from nonurinary source and for which they received active carbapenems (CTG) or BLICs (BTG) as empiric and/or definitive treatment account for at least 50% of total duration of therapy. Exclusion criteria included early deaths (died within 48 h after BSI onset), pregnant, missing key data, and infected strains resistant to BLICs or carbapenems in vitro. Only the first BSI episode for each patient was considered for the analysis.

### Data collection

Data were obtained from LIS and HIS, including patients’ demographic characteristics, microbiological data, source of BSI, severity of illness, record of antibiotic treatment, and clinical outcomes. The collection of individual data starts from the patient’s admission to death or discharge, whichever occurred earlier. The severity of chronic underlying comorbidity was quantified with an age-adjusted Charlson comorbidity index (aCCI), measured on the day of admission. The severity of acute condition was based on Sequential [Sepsis-related] Organ Function Assessment score (SOFA score) and the Pitt bacteraemia score. The SOFA score and Pitt bacteraemia score were measured on the day of BSI onset.

### Definitions and outcomes

*E. coli* or *K. pneumoniae* BSI defined as patients with at least one positive blood culture and developed the following at least two symptoms and signs: (1) body temperature > 38 °C or < 36 °C; (2) heart rate > 90 beats per minute; (3) respiratory rate > 20 breaths per minute; (4) the peripheral blood leukocyte > 10 × 10^9^ /L or < 4 × 10^9^ /L. BSI onset was defined as the day of collection of the first positive blood culture. Source of BSI was established under the guidance of the Centers for Disease Control and Prevention (CDC) criteria [22]. Monomicrobial BSI were defined as the growth of only one microorganism in a blood culture, excluding potential contaminants (e.g., *Propionibacterium *spp*.*, *Corynebacterium *spp*.* and coagulase-negative *staphylococci*).

BSI episodes were classified as nosocomial acquisition when infection symptoms occurred > 2 days of hospital admission and ≤ 2 days after discharge; otherwise, the case was considered community-acquired. Antibiotic treatment was defined as active if the isolate was susceptible or intermediate in vitro. Empiric therapy referred to antibiotic treatment administered before the susceptibility testing report was known; they were considered targeted thereafter. Empirical therapy was defined as “appropriate” if active agent was started within 24 h of initial blood culture collection and continued for at least 48 h, delayed or no active agent was administered during this period was defined as “inappropriate” The treatment duration for bacteraemia was defined as the number of days from commencement to cessation of appropriate antibiotic regimen. Immune compromise refers to the use of large dose corticosteroids (prednisolone or equivalent at least 30 mg/day), antitumor necrosis factor agents, cytotoxic chemotherapy, immunosuppressive therapy (e.g., methotrexate, cyclosporin, tacrolimus everolimus, azathioprine, and mycophenolate), neutrophil count < 500/uL (multiplied by 0.001 to convert to × 10^9^/L) on the day of BSI, or uncontrolled HIV infection (with a CD4 count < 200/mm^3^).

The main outcome variables were the clinical response at day 14 in addition to 30-day mortality after the onset of bacteraemia. Clinical response was dichotomised as cure/improvement versus failure. Clinical cure refers to resolution of all symptoms and signs related to the infection and no further need for antibiotic therapy while improvement was defined as complete or partial resolution of all symptoms and signs related to infection but continued with another antibiotic due to de-escalation. We defined failure as infection-related symptoms or signs that persisted or worsened compared with the one at the BSI onset, or death due to any cause.

### Microbiology

*E. coli* and *K. pneumoniae* isolates were identified using the Vitek 2 Compact system (bioMérieux, France) or MALDI-TOF MS (bioMérieux, France), and antimicrobial susceptibility testing was performed using the VITEK-2 Compact ASTGN16 (bioMérieux, France) or a Kirby–Bauer test. The minimum inhibitory concentration (MIC) was determined according to the Clinical Laboratory Standards Institute (CLSI) criteria for tested antibiotics. ESBL production was tested using the combination disc method in accordance with CLSI protocols using ceftazidime and cefotaxime alone or in combination with clavulanic acid.

### Statistical analysis

Statistical analyses were performed using SPSS 22. For the descriptive analysis, continuous variables were expressed as mean and standard deviation (SD) or median and interquartile range (IQR), depending on whether the distribution of data was normal. Categorical variables were expressed as accumulated frequency and percentage. Groups were compared using the Mann–Whitney U-test or Student’s t-test for continuous variables and the chi-square test or Fisher’s exact test for categorical variables.

To adjust for selection bias in the BLIC and carbapenem treatment groups, we conducted a propensity score matching analysis using 1:2 nearest-neighbour matching without replacement with a calliper length of 0.2. The probability of receiving BLICs or carbapenem therapy was calculated using a logistic regression model, and covariates were identified by comparing patients in the BTG with those in the CTG using univariate analysis. Covariates with P < 0.1 were introduced into the multivariate analysis. The balance between the two groups was analysed by comparing all variables.

Fourteen-day treatment failure, 30-day mortality, and the risk factors for mortality were analysed by Cox regression model using backward elimination, and all variables with P < 0.1 in univariate analysis and potential confounders, were entered into the multivariate model. Results are presented as hazard ratio (HR), 95% confidence interval (CI), and P-values. All P-values were two-sided, and those < 0.05, were considered statistically significant.

## Results

**Fig. 1:**
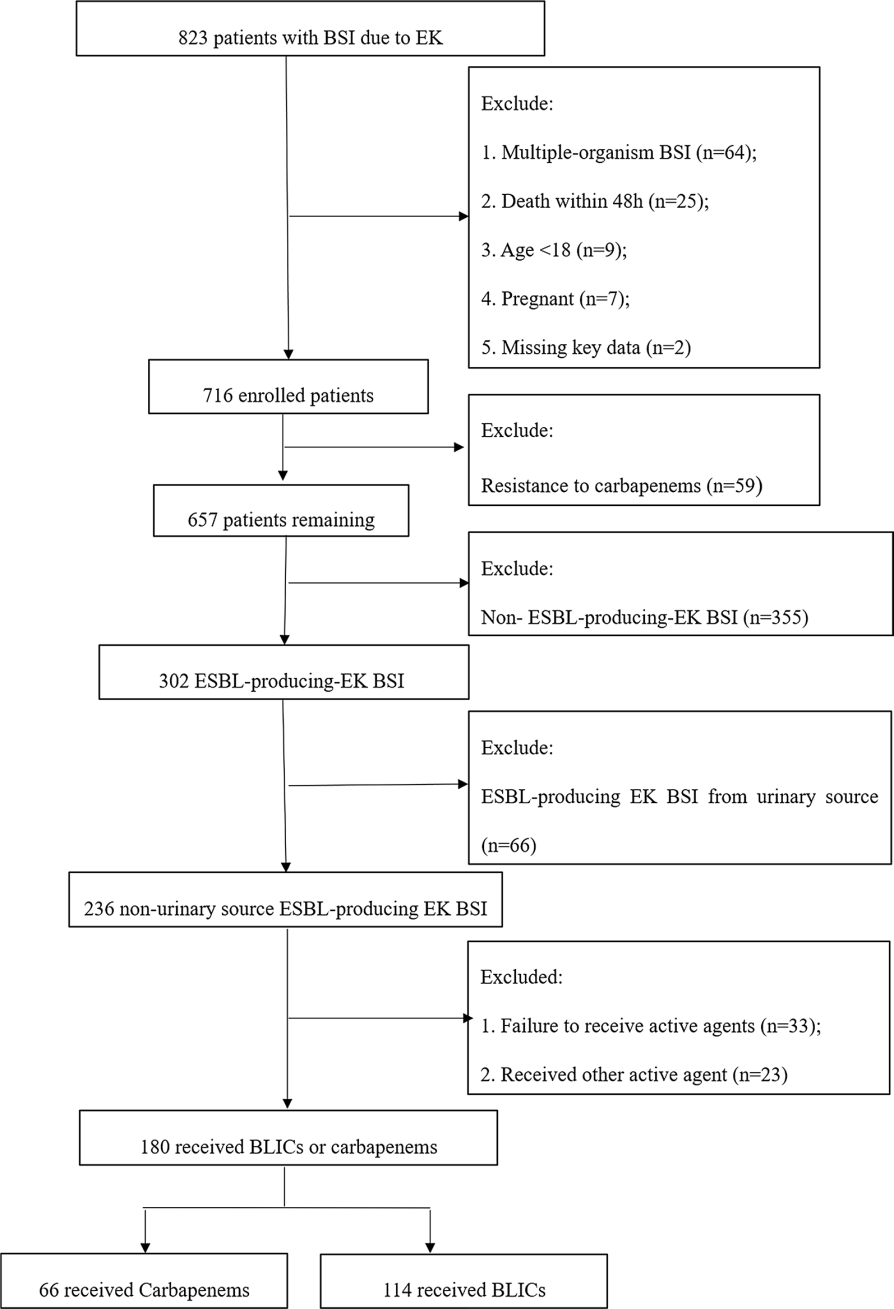
Flow chart of patients selected

During the study period, 823 patients were diagnosed with *E. coli* or *K. pneumoniae* bloodstream infections screened, 180 ESBL-producing EK BSI from nonurinary source were included in the final analysis. Of the 180 patients, 114 received BLIC and 66 received carbapenem therapy, respectively (Fig. 1).

The mean age of the 180 patients was 59.3 years (SD: 13.1), and males accounted for 50.6% (91/180). Hospital-acquired infection occurred in 65% (117/180) of patients, and most patients had hypoproteinaemia (26.1%) and hypertension (23.2%). A total of 81.7% (147/180) of bacteraemia episodes caused by *E. coli.*

When comparing patients treated with BLICs with those treated with carbapenems, we found that BLICs-treated patients were older and had higher aCCI, but shorter length of hospital stay, although they had lower Pitt bacteremia score and SOFA score, lower rate of immune compromise, and leukaemia. After the adjustment of PSM, the baseline characteristics of the two treatment groups were well balanced. The data are presented in Table 1.

**Table 1:** Characteristics of patients

Characteristic	Total	Complete cohort (N = 180)			Cohort adjusted by PSM^a^		
		BLICs	Carbapenems	*P*	BLICs	Carbapenems	*P*
*N* (%)	180	N = 114 (63.3%)	N = 66 (36.7%)		N = 63 (59.4%)	N = 43 (40.6%)	
Age, years							
Mean (SD)	59.3 (13.1)	61.7 (12.4)	55.2 (13.3)	0.001	58.2 (12.3)	55.7 (11.9)	0.469
Sex, *N* (%)							
Male	91 (50.6)	58 (50.9)	33 (50.0)	0.910	30(47.6)	22 (51.2)	0.720
Female	89 (49.4)	56 (49.1)	33 (50.0)		33 (52.4)	21 (48.8)	
Acquisition, *N* (%)							
Hospital-acquired	117 (65.0)	71 (62.3)	46 (69.7)	0.315	41 (65.1)	32 (74.4)	0.308
Community-associated	63 (35.0)	43 (37.7)	20 (30.3)		22 (34.9)	11 (25.6)	
Length, median (IQR)							
Length of hospital stay	20.5 (12–30)	18 (11–28)	24 (14–32)	0.016	20 (11–29)	25 (14–32)	0.055
Severity of condition, median (IQR)							
aCCI	3 (2–5)	4 (2–6)	3 (2–4.25)	0.036	3 (2–5)	3 (2–4)	0.797
Pitt bacteraemia score	1 (0–2.75)	1 (0–2)	2 (1–4)	0.000	1 (0–2)	1 (0–2.5)	0.995
SOFA score	3 (2–6)	3 (1–5)	5 (2.75–9)	0.000	4 (2–6)	3 (1.3–6)	0.517
Underlying diseases, *N* (%)							
Immune compromise	35 (19.4)	15 (13.2)	20 (30.3)	0.005	13 (20.6)	13 (30.2)	0.259
Leukaemia	13 (7.2)	3 (2.6)	10 (15.2)	0.002	3 (4.8)	7 (16.3)	0.086
Arrhythmia	16 (8.9)	11 (9.6)	5 (7.6)	0.638	5 (7.9)	3 (7.0)	1.000
Hypertension	42 (23.2)	27 (23.7)	15 (22.7)	0.884	12 (19.0)	7 (16.3)	0.970
Diabetes	20 (11.1)	14 (12.3)	6 (9.1)	0.512	6 (9.5)	2 (4.7)	0.469
Hypoproteinaemia	47 (26.1)	32 (28.1)	15 (22.7)	0.432	16 (25.4)	9 (20.9)	0.820
Bacteria, *N* (%)				0.448			0.778
*Escherichia coli*	147 (81.7)	95 (83.3)	52 (78.8)		54 (85.7)	36 (83.7)	
*Klebsiella pneumoniae*	33 (18.3)	19 (16.7)	14 (21.2)		9 (14.3)	7 (16.3)	
Empirical treatment, *N* (%)				0.268			0.154
Appropriate	159 (88.3)	103(90.4)	56 (84.8)		60 (95.2)	37 (86.0)	
Inappropriate	21 (11.7)	11 (9.6)	10 (15.2)		3 (4.8)	6 (14.0)	

^a^Adjustment using propensity score matching, covariates including age, length of hospital stay, aCCI, Pitt bacteraemia score, SOFA score, immune compromise, and leukaemia

Of the 63 patients in the propensity-matched cohort of BLICs, 57 received BLICs as appropriate empiric treatment, of which 47 remained on BLICs as definitive therapy, 9 did not receive any antibiotic in definitive treatment, and 1 shifted to another agent but less than 50% of the total treatment duration; 3 patients received BLICs as definitive treatment only; another 3 patients were administered another agent for empirical treatment, however, in definitive therapy, they were all treated with BLICs. BLICs included piperacillin/tazobactam and cefoperazone/sulbactam in 69.8% (44/63) and 30.2% (19/63) of cases, respectively.

Of the 43 patients in the propensity-matched cohort of carbapenems, 31 were treated with carbapenems in empirical therapy, of which 25 continued to receive carbapenems in definitive therapy, 3 did not receive any antibiotic in targeted therapy, and 3 shifted to another antibiotic but for less than half of the total treatment duration; 6 patients received carbapenems as definitive treatment only; while another 6 received another antibiotic in empirical therapy but all shifted to carbapenems for targeted therapy.

After PSM, the 14-day treatment failure rate in BTG (20.6%, 13/63) was higher than that in the CTG (16.3%, 7/43), but the differences were not statistically significant in either the univariate analysis (P = 0.429) or multivariate analysis (HR [95%CI] 2.19 [0.81–5.90], P = 0122). The 30-day mortality rate was similar in both groups: 11.1% (7/63) in BTG and 11.6% (5/43) in CTG. These rates did not significantly differ in univariate analysis (P = 0.926) or multivariate analysis (HR [95%CI] 1.68 [0.48–5.93], P = 0.420). The data are presented in Table 2.

**Table 2:** Outcome of patients treated with BLICs or carbapenems

Outcomes	BLICs		Carbapenems		Univariate analysis		Multivariate analysis	
	*N* (%)	Adjusted *N* (%)	*N* (%)	Adjusted *N* (%)	*P*	Adjusted *P*	HR (95%CI), *P*	Adjusted HR (95%CI), *P*
14-day treatment failure	25 (21.9)	13 (20.6)	17 (25.8)	7 (16.3)	0.970	0.429	1.61 (0.74–3.49), 0.230	2.19 (0.81–5.90), 0.122
30-day mortality	13 (11.4)	7 (11.1)	13 (19.7)	5 (11.6)	0.289	0.926	1.57 (0.62–4.01), 0.345	1.68 (0.48–5.93), 0.420

The risk factors for 30-day mortality in BTG and CTG are shown in Tables 3 and 4, respectively. After adjusting for selection bias, the multivariate model showed that leukaemia was the only independent predictor of 30-day mortality in both BTG (HR [95%CI] 7.79 [1.48–40.86], P = 0.015) and CTG (HR [95%CI] 11.35 [1.17–109.83], P = 0.036).

**Table 3:** Risk factors for 30 day mortality in patients treated with BLICs

	Univariate analysis	Multivariate analysis	Univariate analysis	Multivariate analysis
Variables	*P*	HR (95%CI), *P*	Adjusted *P*	Adjusted HR (95%CI), *P*
Leukemia	0.018	8.82 (1.79–43.33), 0.007	0.015	7.79 (1.48–40.86), 0.015
SOFA score	0.037	1.21 (0.98–1.50), 0.074	0.150	1.28 (0.93–1.77), 0.132
aCCI	0.033	1.22 (1.03–1.45), 0.019	0.280	
Pitt bacteremia score	0.300		0.242	
Age	0.713		0.959	
Immune-compromise	0.981		0.659

**Table 4:** Risk factors for 30 day mortality in patients treated with carbapenems

	Univariate analysis	Multivariate analysis	Univariate analysis	Multivariate analysis
Variables	*P*	HR (95%CI), *P*	Adjusted *P*	Adjusted HR (95%CI), *P*
Age	0.021	0.94 (0.90–0.98), 0.005	0.254	
Leukemia	0.064	8.64 (1.60–46.47), 0.012	0.036	11.35 (1.17–109.83), 0.036
Pitt bacteremia score	0.320	1.45 (1.08–1.96), 0.014	0.969	
aCCI	0.640		0.794	
SOFA score	0.731		0.462	0.74 (0.38–1.44), 0.378

## Discussion

Out of 823 infected patients, 180 had infection due to ESBLs producers. In China, *E. coli* is the predominant ESBL producer, and the antimicrobial resistance surveillance programme showed that the prevalence of ESBL-producing *E. coli* increased from 38.9% in 2005 to 55.8% in 2014, while that of *K. pneumoniae* has declined (decreased from 39.1 to 29.9%), but it is still maintained at a high level [23]. The high prevalence of ESBL among Enterobacteriaceae mostly due to overuse of antibiotic, non-adjusted course of the drug or uncompleted dosage and inappropriate antibiotic administration [24]. On the other hand, BLICs and carbapenems are the most common agents used in patients with severe infections caused by ESBLs producers [9, 25]. Therefore, to reduce unsuitable antibiotic administration and bring positive effects on antibiotic stewardship, it is necessary to evaluate the efficacy of the two drugs.

In this study, we found that BLICs had a higher treatment failure rate at day 14 for patients with BSI from nonurinary source caused by ESBL-producing EK, although the statistical differences were not significant, which affected by the small number of patients.

Over the past decade, *β*-lactam/*β*-lactamase inhibitor combinations have become the most interesting alternatives to carbapenems used in patients with infections caused by ESBL-producing Enterobacteriaceae [8]. Despite the fact that BLICs have a broad antibacterial spectrum, good bactericidal activity, low tendency to select *Clostridium difficile* infections, and further antibiotic resistance [9, 26, 27], the significance of BLICs for treating ESBL-producing Enterobacteriaceae has remained controversial.

A multinational, random clinical trial found that for patients with BSI due to ceftriaxone resistance *E coli* or *K pneumoniae* bloodstream infection, the 30-day mortality rate in the cohort of piperacillin/tazobactam (12.3%, 23/187) was significantly higher than meropenem (3.7%, 7/191) (P = 0.90 for noninferiority) [5]. While a classic, frequently quoted study has indicated that for BSI due to ESBL-producing *Escherichia coli* piperacillin/tazobactam are suitable alternatives to carbapenems [18]. However, the participants in that study had BSI mostly originated from urinary (or biliary) tract and the number of critically ill patients with high bacterial loads was likely to be low. Notably, Harada et al. [28] proved that piperacillin/tazobactam is less effective both in vitro and in vivo when tested against high inoculum infection. Therefore, it is reasonable to suspect that the high proportion of BSI episodes from urinary source in that study is the main reason for the different results from ours.

Our data revealed an intriguing phenomenon that before PSM (no statistical differences in patient outcomes) patients treated with BLICs with older age and higher aCCI but had a shorter length of hospital stay, although their acuity of condition at BSI onset (reflected by higher Pitt bacteraemia score and SOFA score), rates of immune compromise and leukaemia were lower than those of patients treated with carbapenems. After PSM, age and aCCI were not related to 30-day mortality (P = 0.959, P = 0.280), the data may send a practical message to clinicians that old age and high burden of chronic illness are not enough to be the main reasons for avoiding BLIC treatment.

In view of the fact that few patients (7.9%, 5/63) matched the stratification criteria of high-risk disease (Pitt bacteraemia score > 4), which may have a tendency to bias the severity of illness to a lower estimate of the risk factor of mortality. Therefore, the use of BLICs in more severe conditions in patients with BSI remains to be discussed. A large randomised clinical trial performed in nine countries indicated that irrespective of infection severity, piperacillin/tazobactam could not result in non-inferiority compared to meropenem [5]. Another large-scale trial conducted by Bertolini indicated that the use of piperacillin/tazobactam fared worse in severely ill patients with extended-spectrum cephalosporin-resistant, but carbapenem-susceptible *Klebsiella spp.* Infection [29]. In general, these two trials indicated that piperacillin/tazobactam should not be used in critically ill patients infected with ESBL-producing EK.

Our results also showed that leukaemia was an independent predictor of all-cause 30-day mortality in patients treated with both BLICs and carbapenems. Bloodstream infection remains an important cause of morbidity and mortality in patients with haematologic malignancies [30, 31], and these patients have a high risk of bacteraemia during chemotherapy, mostly due to immune suppression and damage of mucosal barriers that facilitate bacterial translocation to the bloodstream [32, 33]. The primary method to prevent infections during chemotherapy has been antibiotics, but utilisation of antibiotics is the major cause of dysbiosis by putting high antibiotic pressure on patients undergoing therapy, avoiding the use of broad-spectrum antibiotics to prevent infections in patients with leukaemia may reduce the severity of dysbiosis and prevent infectious complications [32, 34]. Actually, even in patients with infection, empirical treatment should not unconditionally use broad-spectrum agents to cover ESBL producers to avoid further selection of bacterial resistance [35]. However, the incidence of colonisation and infection by ESBL-producing Enterobacteriaceae is high in patients with malignancy [36, 37], and delays in appropriate empirical treatment may be associated with poor outcomes [35]. Given all these, previous screening for ESBL-producing bacteria colonisation in such high-risk patients to guide the use of antibiotics may be an effective measure to improve patient outcomes.

The data should be interpreted carefully in our study from several aspects. First, this was a single-centre study; therefore, the ability to follow patients and complete missing data was limited, and the results may have been influenced by local epidemiology and prescription practices. Second, although we conducted propensity score matching and multivariate analysis to adjust for selection bias and potential confounders, adverse effects from non-matched confounding factors could remain. Third, caution should be exercised when generalising the results of this study to other ESBL-producing Enterobacteriaceae infections, because most patients in our cohort had BSI due to *E. coli*, which is usually related to better outcomes than other Enterobacteriaceae [15, 38].

## Conclusion

Among patients with ESBL-producing EK BSI of nonurinary source, compared with carbapenem, BLIC treatment had higher 14-day treatment failure rate, we do not support use of BLICs in this setting. More retrospective or prospective studies are needed to further assess the efficacy of BLICs.

## Data Availability

All the data are from the database of the second affiliated Hospital of Nanchang University. The raw data supporting the conclusions of this manuscript will be made available by the authors, without undue reservation, to any qualified researcher.

## Abbreviations

ESBL-producing EK: Extended-spectrum β-lactamase-producing *E. coli* or *K. pneumoniae*
BLICs: *β*-Lactam/*β*-lactamase inhibitor combinations
BSI: Bloodstream infections
aCCI: Age-adjusted Charlson comorbidity index
SOFA score: Sequential [Sepsis-related] Organ Function Assessment score
PSM: Propensity score matching
BTG: BLIC-treated group
CTG: Carbapenem-treated group
LIS: Laboratory Information System
HIS: Hospital Information System
MIC: Minimum inhibitory concentration
CLSI: Clinical Laboratory Standards Institute
SD: Standard deviation
IQR: Interquartile range
HR: Hazard ratio
CI: Confidence interval
